# Genomic Sequences and Analysis of Five SARS-CoV-2 Variants Obtained from Patients in Lambayeque, Peru

**DOI:** 10.1128/MRA.01267-20

**Published:** 2021-01-07

**Authors:** Franklin Rómulo Aguilar-Gamboa, Luis Alberto Salcedo-Mejía, Luis Miguel Serquén-López, Marco Enrique Mechan-Llontop, Percy Omar Tullume-Vergara, Juan José Bonifacio-Briceño, Ramsés Salas-Asencios, Heber Silva Díaz, Juan P. Cárdenas

**Affiliations:** a Dirección de Investigación, Laboratorio de Inmunología–Virología, Hospital Regional Lambayeque, Lambayeque, Peru; b Laboratorio de Biotecnología, Facultad de Ciencias Naturales y Matemática, Universidad Nacional Federico Villarreal, Lima, Peru; c Department of Microbiology and Molecular Genetics, Michigan State University, East Lansing, Michigan, USA; d Department of Parasitology, Institute of Biological Science, University of São Paulo, São Paulo, Brazil; e Grupo de Investigación Bienestar y Salud Global, Escuela Universitaria de Posgrado, Universidad Nacional Federico Villarreal, Lima, Peru; f Center for Genomics and Bioinformatics, Faculty of Sciences, Universidad Mayor, Santiago, Chile; g School of Biotechnology, Faculty of Sciences, Universidad Mayor, Santiago, Chile; h Umbrella Genomics Company S.A.C., Lima, Peru; DOE Joint Genome Institute

## Abstract

Here, we report the genomic sequences of five severe acute respiratory syndrome coronavirus 2 (SARS-CoV-2) strains obtained from nasopharyngeal samples from five tested coronavirus disease 2019 (COVID-19)-infected patients from the Lambayeque region in Peru during early April 2020.

## Abstract (precis)

Here, we report the genomic sequences of five severe acute respiratory syndrome coronavirus 2 (SARS-CoV-2) strains obtained from nasopharyngeal samples from five tested COVID-19-infected patients from the Lambayeque region in Peru during early April 2020.

## ANNOUNCEMENT

Here, we present the genomes of five severe acute respiratory syndrome coronavirus 2 (SARS-CoV-2) (family *Coronaviridae*; genus *Betacoronavirus*; subgenus *Sarbecovirus*) variants sequenced from five patients from whom samples were taken in different districts in the Lambayeque region of Peru (La Victoria, Monsefú, Chiclayo, José Leonardo Ortiz, and Lambayeque), during early April 2020.

Ethical approval for sample recollection and analysis protocols was given by the ethics committee of the Regional Lambayeque Hospital (code 0212-028-20CEI). This study has been registered in Proyectos de Investigación en Salud, del Instituto de Salud del Perú (PRISA; dependent on the National Health Ministry, Peru), with code 37EBF149-F123-447F-8970-680BF549DE1D.

Nasopharyngeal swabs from SARS-CoV-2-positive patients (cycle threshold [*C_T_*] values obtained by qPCR, <25) were sampled, and total RNA was isolated using the GenUP total RNA kit (BiotechRabbit, Germany). rRNA depletion was carried out using the Ribozero rRNA removal kit (Illumina). The cDNA libraries were generated using the TruSeq stranded total RNA LT kit (Illumina), using a single index and a fragment size range of 388 to 523 bp. Sequencing for every sample was performed in an Illumina NextSeq 500 model (midoutput 300 cycles), generating paired-end reads (150 bases long). Illumina sequencing was performed at Genoma Mayor (Universidad Mayor, Chile).

Raw paired-end reads were processed by using Trimmomatic (1) version 0.39, generating trimmed 129-bp reads. Viral genomes were assembled using a reference-guided approach, using the Wuhan Hu-1 strain genome (accession number MN908947) as a reference. Read mapping was done using SAMtools version 1.10 (2); mapped reads were analyzed by using Trinity (3) version 2.1.1 in the mode “--genome_guided_bam,” obtaining near-full-length genomes (shortest assembly length, 29,854 bp). Genomes were annotated with the RATT tool (4), using the annotation of MN908947 as a reference.

Sequence variants were checked by two approaches for each sample: the first one was to detect the variants from the consensus assemblies, by the use of the global alignment tool *needle* from the EMBOSS package (5), comparing each assembly against the aforementioned reference. The second strategy was made from read mapping and single-nucleotide polymorphism (SNP) variant calling in comparison with the reference by the use of the tool bcftools call (6) version 1.7, using the parameters “--ploidy 1 -c.” Both strategies gave the same results. Their incomplete 5′ and 3′ ends (trimmed due to very low sequence coverage) did not affect the completeness of any coding sequences (CDS). Therefore, all assemblies were technically complete according to GenBank standards.

In order to classify these Peruvian variants, genomes were analyzed by using different tools. According to PANGOLIN (Phylogenetic Assignment of Named Global Outbreak LINeages) (7), all five variants were classified in the B.1.1.1 lineage. According to the NextClade tool from Nextstrain (8), all variants were classified in the 20B clade. Those PANGOLIN and Nextstrain clusters were previously reported as containing representative groups of sequenced Peruvian SARS-CoV-2 strains (9). According to the aforementioned sequence variant process, there are between 12 and 14 mutations for each strain (Table 1), including the previously described mutation P323L in Nsp12 (P4715L from ORF1b) and D614G in the spike protein, and such variants are becoming increasingly abundant worldwide (10).

**TABLE 1:** Main sequencing statistics and features found in the five SARS-CoV-2 assemblies^*a*^

Parameter			Result for sample:				
			HRL-235	HRL-239	HRL-187	HRL-223	HRL-224
GISAID identifier			EPI_ISL_593772	EPI_ISL_593773	EPI_ISL_593774	EPI_ISL_593775	EPI_ISL_593776
Total no. of raw reads			53,033,958	35,123,841	26,161,830	26,717,077	31,546,940
Total no. of QC-passed reads			35,081,171	16,845,649	18,732,847	21,659,368	193,21,178
Mapped no. of reads (against reference)			2,243,450	30,147	194,144	1,252,518	1,970,766
Avg coverage depth (×) (against reference)			5,951.51	129.696	837.031	4,386.75	5,670.8
% GC content			42	43	44	44	42
Evidence (X) of:							
Mutation	Gene/region	Mutation type					
C241T	5′ UTR	NA	X	X	X	X	X
C3037T	ORF1a	Silent	X	X	X	X	X
C4002T	ORF1a	T1246I	X	X	X	X	X
G10097A	ORF1a	G3278S	X	X	X	X	X
A10323G	ORF1a	K3353R			X	X	
C13536T	ORF1a	Silent	X	X	X	X	X
C14408T	ORF1b	P4715L	X	X	X	X	X
T14775C	ORF1b	Silent		X			
A23403G	S	D614G	X	X	X	X	X
C23731T	S	Silent	X	X	X	X	X
C24442T	S	Silent		X			
G27160T	M	S213I				X	
G28881A	N	R203K	X	X	X	X	X
G28882A	N	R203K	X	X	X	X	X
G28883C	N	G204R	X	X	X	X	X
G29179T	N	Silent	X	X	X	X	X

a QC, quality checked; UTR, untranslated region; NA, not applicable.

### Data availability.

The sequences were deposited in GISAID with the codes EPI_ISL_593772, EPI_ISL_593773, EPI_ISL_593774, EPI_ISL_593775, and EPI_ISL_593776. Assembled genomic sequences were deposited in GenBank under accession numbers MW185823, MW185824, MW185825, MW185826, and MW185827. Illumina raw reads for this sequencing project (PRJNA673055) can be accessed under accession numbers SRR12927887, SRR12927888, SRR12927889, SRR12927890, and SRR12927891.

## References

[1] Bolger AM, Lohse M, Usadel B. 2014. Trimmomatic: a flexible trimmer for Illumina sequence data. Bioinformatics 30:2114–2120. doi:10.1093/bioinformatics/btu170. 24695404 PMC4103590

[2] Li H, Handsaker B, Wysoker A, Fennell T, Ruan J, Homer N, Marth G, Abecasis G, Durbin R, 1000 Genome Project Data Processing Subgroup. 2009. The Sequence Alignment/Map format and SAMtools. Bioinformatics 25:2078–2079. doi:10.1093/bioinformatics/btp352. 19505943 PMC2723002

[3] Haas BJ, Papanicolaou A, Yassour M, Grabherr M, Blood PD, Bowden J, Couger MB, Eccles D, Li B, Lieber M, MacManes MD, Ott M, Orvis J, Pochet N, Strozzi F, Weeks N, Westerman R, William T, Dewey CN, Henschel R, LeDuc RD, Friedman N, Regev A. 2013. De novo transcript sequence reconstruction from RNA-seq using the Trinity platform for reference generation and analysis. Nat Protoc 8:1494–1512. doi:10.1038/nprot.2013.084. 23845962 PMC3875132

[4] Otto TD, Dillon GP, Degrave WS, Berriman M. 2011. RATT: rapid annotation transfer tool. Nucleic Acids Res 39:e57. doi:10.1093/nar/gkq1268. 21306991 PMC3089447

[5] Rice P, Longden I, Bleasby A. 2000. EMBOSS: the European Molecular Biology Open software suite. Trends Genet 16:276–277. doi:10.1016/s0168-9525(00)02024-2. 10827456

[6] Danecek P, McCarthy SA. 2017. BCFtools/csq: haplotype-aware variant consequences. Bioinformatics 33:2037–2039. doi:10.1093/bioinformatics/btx100. 28205675 PMC5870570

[7] Rambaut A, Holmes EC, O’Toole Á, Hill V, McCrone JT, Ruis C, Du Plessis L, Pybus OG. 2020. A dynamic nomenclature proposal for SARS-CoV-2 lineages to assist genomic epidemiology. Nat Microbiol 5:1403–1407. doi:10.1038/s41564-020-0770-5. 32669681 PMC7610519

[8] Hadfield J, Megill C, Bell SM, Huddleston J, Potter B, Callender C, Sagulenko P, Bedford T, Neher RA. 2018. Nextstrain: real-time tracking of pathogen evolution. Bioinformatics 34:4121–4123. doi:10.1093/bioinformatics/bty407. 29790939 PMC6247931

[9] Juscamayta-López E, Tarazona D, Valdivia F, Rojas N, Carhuaricra D, Maturrano L, Gavilán R. 2020. Phylogenomic reveals multiple introductions and early spread of SARS-CoV-2 into Peru. BioRxiv doi:10.1101/2020.09.14.296814. PMC8426889 34185310

[10] Ilmjärv S, Abdul F, Acosta-Gutiérrez S, Estarellas C, Galdadas I, Casimir M, Alessandrini M, Gervasio FL, Krause K-H. 2020. Epidemiologically most successful SARS-CoV-2 variant: concurrent mutations in RNA-dependent RNA polymerase and spike protein. medRxiv doi:10.1101/2020.08.23.20180281. PMC8249556 34210996

